# Local search for the generalized tree alignment problem

**DOI:** 10.1186/1471-2105-14-66

**Published:** 2013-02-26

**Authors:** Andrés Varón, Ward C Wheeler

**Affiliations:** 1 Division of Invertebrate Zoology, American Museum of Natural History, New York, NY - 10024, USA

**Keywords:** Tree alignment, Tree search, Phylogeny, Sequence alignment, Direct optimization

## Abstract

**Background:**

A phylogeny postulates shared ancestry relationships among organisms in the form of a binary tree. Phylogenies attempt to answer an important question posed in biology: what are the ancestor-descendent relationships between organisms? At the core of every biological problem lies a phylogenetic component. The patterns that can be observed in nature are the product of complex interactions, constrained by the template that our ancestors provide. The problem of simultaneous tree and alignment estimation under Maximum Parsimony is known in combinatorial optimization as the Generalized Tree Alignment Problem (GTAP). The GTAP is the Steiner Tree Problem for the sequence edit distance. Like many biologically interesting problems, the GTAP is NP-Hard. Typically the Steiner Tree is presented under the Manhattan or the Hamming distances.

**Results:**

Experimentally, the accuracy of the GTAP has been subjected to evaluation. Results show that phylogenies selected using the GTAP from unaligned sequences are competitive with the best methods and algorithms available. Here, we implement and explore experimentally existing and new local search heuristics for the GTAP using simulated and real data.

**Conclusions:**

The methods presented here improve by more than three orders of magnitude in execution time the best local search heuristics existing to date when applied to real data.

## Background

A phylogeny postulates shared ancestry relationships among organisms in the form of a binary tree. Phylogenies attempt to answer an important question posed in biology: what are the ancestor-descendent relationships between organisms? At the core of every biological problem lies a phylogenetic component. The patterns that can be observed in nature are the product of complex interactions, constrained by the template that our ancestors provide. For example, the presence and structure of the human skull is mainly determined by its structure in our ancestors. The relationship between the features observed in different organisms can only be understood if the phylogenetic relationships can be hypothesized.

An important method of phylogenetic inference is Maximum Parsimony (MP). Under MP, the preferred hypothesis is the one that minimizes the number of evolutionary transformations required to explain the observed features [1]. This optimization problem is known in Computer Science as the Steiner Tree problem, which is NP-Complete [2].

The problem of *simultaneous tree and alignment estimation* under Maximum Parsimony is known in combinatorial optimization as the Generalized Tree Alignment Problem (GTAP) [3]. The GTAP is the Steiner Tree Problem for the sequence edit distance. Like many biologically interesting problems, the GTAP is NP-Hard [2]. Typically the Steiner Tree is presented under the Manhattan or the Hamming distances. (We will refer to these two forms generically as the STP.) Experimentally, the accuracy of the GTAP has been subject to evaluation [4-6]. The most recent results have shown evidence that phylogenies selected using the GTAP from unaligned sequences are competitive (in terms of optimal and accurate solutions) with the best methods and algorithms available based on coupled, but separate multiple sequence alignment and phylogeny reconstruction [5,6].

Due to its computational hardness, biologists interested in the GTAP rely on heuristic procedures to find good solutions. The simplest, and arguably the most important heuristic for the GTAP is a *local search*. A local search iteratively evaluates trees similar to a current solution *T*, where similar trees constitute the neighborhood of *T*. If a shorter tree *S* is found in the neighborhood, then *T* is replaced by *S*, and the search continues. Otherwise, *T* is the final solution. Local search is the work horse of most phylogenetic analysis procedures of practical use, and the core search procedures to solve the GTAP in the computer programs MSAM [7], and POY [8,9]. It is known that the quality of a GTAP analysis is heavily dependent on the fit of the local search heuristics used [5], but the question of which heuristics are a better fit under what conditions remains unanswered.

In this paper, we discuss, implement, and experimentally explore existing and new local search heuristics for the GTAP using simulated data. Our methods improve by more than three orders of magnitude the best local search heuristics existing to date with real data. We begin by formally explaining the existing heuristics, and new heuristics for the GTAP. Following the results of [9], we use the Affine-DO algorithm to compute the tree length heuristically.

## The algorithms

A subproblem of the GTAP is the Tree Alignment Problem (TAP) (see [10]). Heuristically solving the TAP with Affine-DO [10] can be done in *O*(*n*^2^|*V*|), where *n* is the maximum sequence length and *V* the vertex set of the tree, and typically *n*≫|*V*|. To simplify notation, in this section we assume that calculating the assignment of a vertex in a tree is a constant time operation (i.e. the score of a tree is computed in *O*(|*V*|) time).

### Existing heuristics

**Figure 1:**
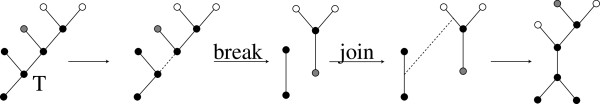
**Breaking and joining a tree.** Breaking a tree in two connected components, and joining them again with a different edge. The resulting tree is part of T’s TBR neighborhood.

A local search consists of two steps: initial tree construction, and refinement (defined below). Given an initial tree *T*, refinement evaluates trees similar to *T*, in the search for a better solution. Those trees similar to *T* are its neighborhood. The most commonly used neighborhood function is known as Tree Bisection and Reconnection (TBR) [11]. TBR is based on two simple tree modifications: breaking an unrooted tree in two components, and joining two separate trees in one (Figure 1): ***Tree Breaking.*** Given a tree *T*, remove an edge (*u*,*v*) to produce two connected components, one with *u*, the other with *v*. If *u* (*v*) is not a leaf, then collapse it.

***Tree Joining.*** Let *T*=(*V*,*E*) and *S*=(*V*^′^,*E*^′^) be two binary trees. *T* and *S* can be joined by selecting a pair of edges (*u*,*v*)∈*E* and (*u*^′^,*v*^′^)∈*E*^′^, create subdivision vertices *x* in the edge (*u*,*v*) and *x*^′^ in (*u*^′^,*v*^′^), and add the edge (*x*,*x*^′^). If *T* (*S*) does not have edges, but only one vertex *v*, then take *v* as *x* (*x*^′^).

The TBR neighborhood of *T* is the set of trees that can be produced by breaking *T* at any edge to produce two trees *U* and *V*, and then joining *U* and *V*. This neighborhood is used in the local search step of the GTAP solver programs POY [8,9] and MSAM [7].

The most popular strategy for the initial tree construction is the *Wagner algorithm*[12], a randomized, greedy strategy, of time complexity *O*(|*V*|^2^). The Wagner algorithm is used in most software packages for phylogenetic analysis under MP (e.g. [13]), including POY [8,9]. MSAM takes a different approach, by using a Neighbor Joining tree, with time complexity *O*(|*V*|^3^) [7]. Deterministic algorithms are not typically used in the tree building step: for non trivial data sets, a good randomized method can be used repeatedly to initiate independent refinements resulting in different solutions. Their shared properties can give insights into the problem’s structure, and help discover better solutions.

Depending on the distance function, different procedures are used to compute the score of the trees in the TBR neighborhood efficiently [14-21]. In particular, for the Hamming and Manhattan distance, to calculate all of the tree scores in the TBR neighborhood has time complexity *O*(|*V*|^3^) [11]. For the GTAP however, it has time complexity *O*(|*V*|^4^) [19,22-24], or *O*(|*V*|^3^) by increasing the hidden factor from *O*(*n*^2^) to *O*(*n*^3^) (remember that typically *n*≫|*V*|) [20,23].

Exploring a neighborhood requires two additional criteria: the stopping rule, and the selection of the next candidate solution. Depending on their properties, a number of local search strategies can be described. A classic heuristic that specifies the stopping and selection criteria is simulated annealing (SA) [25-27]. Contradictory conclusions about the applicability of SA to phylogenetic analysis can be found in the literature [18,26-29]. A form of simulated annealing with better performance under the Hamming and Euclidean distance is known as Tree-Drifting [18]. However, its Metropolis and stopping criteria make Tree-Drifting inapplicable to the GTAP. The potential of Simulated Annealing for the GTAP has remained unexplored.

Sectorial search [18] (SS) is a heuristic that restricts or extends the TBR neighborhood by only breaking and joining selected subtrees (i.e. connected subgraphs), or exhaustively solving such subtrees. Two variations of this scheme have been proposed: in the Random-Based SS, subtrees are selected uniformly at random. In the second variation, the Consensus-Based SS, given a parameter 0≤*n*≤1, only rearrange (or evaluate exhaustively) subtrees occurring in at least *n*∗*m* solutions found in *m* previous searches (*n* typically set to 0.85) [18].

Other strategies (e.g. Parsimony Ratchet, Tree Fusing, the Genetic Algorithm, DCM), do not strictly belong to the set of local search heuristics. Given that local search is part of all these strategies, all of them would be more efficient if a good local search is in place.

### New heuristics for the GTAP

In this section, we describe four ideas to improve the local search strategies in the GTAP: efficient tree length calculation during the search, better tree cost bounding, a smarter local search strategy, and initial tree building algorithms.

#### Efficient tree updates

To apply the selection and stopping rules during TBR, it is necessary to calculate the tree length after every break, and join. Affine-DO requires a *directed* tree as induced by its root. If the sequence edit distance function is metric, the true tree length is independent of the root location. Given that metric distances are a common requirement under MP we assume from now on that the edit distance is metric. It follows that, although Affine-DO can produce a different tree length for each possible root, there is no constraint to maintain one.

**Figure 2:**
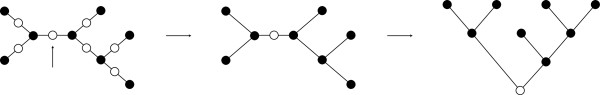
**All possible roots of an unrooted tree.** All possible roots of the unrooted tree correspond to the subdivision vertices of its edges (empty circles).

To update a tree efficiently, we do not maintain a unique rooted representation, but rather take its unrooted representation and keep all the potential roots assigned to every edge of the tree (Figure 2). We call this a *three directional assignment*. Although we describe it for its application for the GTAP, it is applicable to any algorithm that requires post-order traversal to compute (or estimate) the tree length. (We have used it successfully under the breakpoint [30], inversion [31], and double cut and join [32] distances.)

##### Three directional assignment

**Figure 3:**
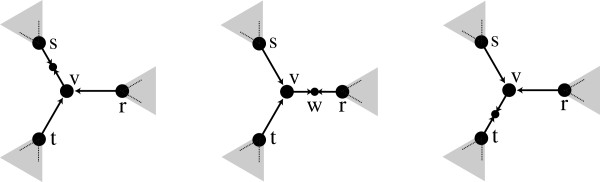
**Three directional assignment.** Three possible assignments to interior vertices of an unrooted tree. Left: computing the subdivision vertex of (*s*,*v*), or any edge rooted by *s* (grey triangle on *s*), would require to compute the assignment to *v* using those of *t* and *r*. Center and right: similarly, the assignment of *v* could be computed using *s* and *t*, or *t* and *r*. Each direction is needed for some subdivision vertices.

For an unrooted binary tree, we assign to each edge (*u*,*v*) a sequence. This sequence is the Affine-DO assignment to the subdivision vertex *w* of (*u*,*v*). Computing Affine-DO(*w*) is dependent on the assignment to its neighbors (Figure 3, center). In a binary tree, each interior vertex has three incident edges. Therefore, there are three possible Affine-DO assignments for every interior vertex (i.e. vertex *v* in Figure 3). Each assignment is required to compute some subdivision vertices. Hence, we maintain the three possible assignments for each interior vertex. These assignments can be computed with time complexity *O*(|*V*|), using first a pre-order traversal then followed by a post-order traversal, starting on any edge.

###### Observation 1

A tree with a three directional assignment computes the length of every tree that can be produced by breaking any one edge with time complexity *O*(|*V*|).

###### Observation 2

Given two separate trees *S* and *T* with the three directional assignment, computing the length of all the trees produced by joining every pair of edges in *S* and *T* has time complexity *O*(|*V*|^2^).

The simplest implementation of the three directions is to eagerly compute all the assignments in preparation for the first tree break, and join. However, such an algorithm would entail overhead for greedy heuristics such as simulated annealing, where the first acceptable tree should be chosen to continue with the local search.

We solve this problem by using lazy evaluation and memoization [33] as follows: eagerly assign a lazy function to each vertex and edge of the tree, but only compute its value (and the values it depends on) upon request, while memoizing the result. In this way, we only spend time computing each vertex if used. This technique has greater value if the tree break, and join order is carefully chosen. In the following section, we will see how the three directional assignment can also be used to improve the estimation of each tree cost with no additional time complexity.

#### Multiple heuristic TAP solutions

The Affine-DO algorithm may calculate different tree length bounds depending on the root location (i.e. one per subdivision vertex). Nevertheless, the best of all the assignments is preferable for each tree. Computing all of the Affine-DO tree lengths, however, would add a *O*(*n*) time complexity multiplicative factor to each tree break and join. We avoid such factor and still produce better bounds for the tree cost during the search by using Algorithm 1 on each break, and Algorithm 2 on each join of the local search.

### Algorithm 1: Improving the bound of a tree on each edge break

### Algorithm 2: Improving the bound of a tree on each join

For a fixed *n*, the join procedure adds only a constant multiplicative factor, without increasing the time complexity. Note that if all the edges of a tree *T* are broken during a local search, then 2*n*−3 alignments are evaluated for the final tree, with no additional time complexity. We call this variation of the TBR Exhaustive-TBR.

#### Smarter local searches

Affine-DO [10] defines a compact representation of sets of sequences called a reduced alignment graph (RAG). RAGs are less powerful than alignment graphs [34], but are simpler and more efficient to compute and use. It is then possible to align RAG’s, find the closest sequences contained in them, and compute their RAG with time complexity *O*(*n*^2^), the same of a regular pairwise sequence alignment [35]. Ultimately, Affine-DO is a method to compute the distance between the closest sequences contained in a pair of RAGs efficiently.

**Figure 4:**
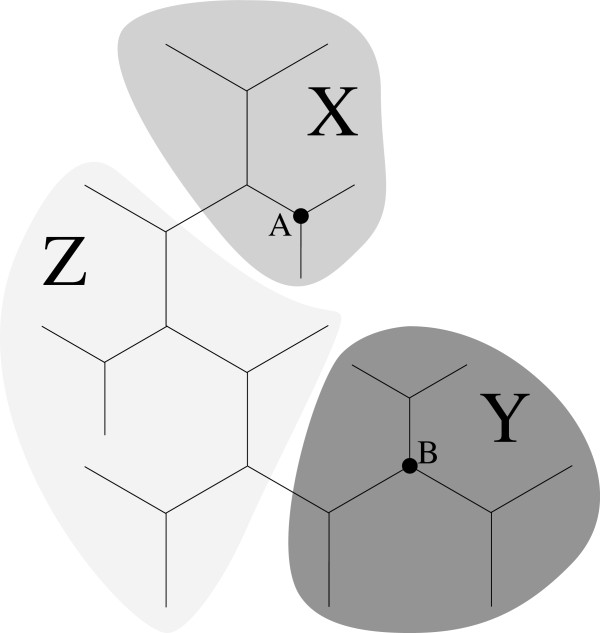
**Unions to bound the cost of a tree.** Use of unions to bound the cost during a local search. Shade areas enclose disjoint sets of vertices in the tree. Suppose that we merge all the RAG’s of each vertex set using Algorithm 3 to produce the unions X, Y, and Z. Then we can heuristically bound *d*(*A*,*B*) as *d*(*X*,*Y*)≤ min*A*∈*C*,*B*∈*Dd*(*A*,*B*), where *d* is the distance as calculated using the Affine-DO alignment algorithm.

RAGs can be used to guide a local search. If the union of a pair of RAGs *A* and *B* can be efficiently computed in a new RAG *C*, then *C* can be used to bound the distance between any other RAG *D* and *A* or *B* simultaneously. Therefore, it is possible to use the union of multiple RAGs assigned to multiple vertices in a tree, to compute a lower bound of the closest pair of sequences contained in a pair of vertex sets (Figure 4).

### Algorithm 3: Algorithm to compute *merge(i, j, k, result)*. The union of a single RAG *A* is 〈*A*_*i*_,*true*〉

#### Theorem 1

Let *R*=*merge*(|*X*|,|*Y*|,|*M*|,〈〉) (Algorithm 3). All the sequences contained in *X*, *Y*, and *M* are contained in *R*.

#### Proof

At each step, either *X*_*i*_, *Y*_*j*_, *M*_*k*_, {*indel*}, or any of their combinations is prepended to the result. Therefore, no element appearing in *X*, *Y*, or *M* is missing in *R*. Moreover, for all 0<*e*,*f*≤|*X*|, *X*_*e*_ is prepended before *X*_*f*_ if and only if *e*<*f*. Hence, the relative order of the elements in *X* is maintained in *R*. Finally, for all the cases where *X*_*i*_ is not prepended, then the *indel* element is included in *R*. It follows that that we can recover *X* by removing those elements in *R* where such indels where inserted and no element of *X* was. By the definition of sequences contained in a RAG [10], it follows that every sequence in *X* is contained in *R*.

The analysis of *Y* and *M* is symmetric. □

#### Theorem 2

Algorithm 3 computes the union of *X*, *Y*, and *M* with time complexity *O*(|*X*|) where *X* is the longest union.

#### Proof

The algorithm stops when *i*,*j*,*k*<1. At each recursive step, either *i* or *j* is reduced by one, with initial values *i*=|*X*| and *j*=|*Y*|. □

The union of RAGs can be executed in *O*(*n*|*V*|), on each vertex, during the Affine-DO computation. Affine-DO is *O*(*n*^2^), therefore, this method entails a small additive factor to the time complexity of Affine-DO. In our implementation, we have fixed the size of the vertex sets to 12 vertices on all data sets experimentally.

#### 

##### Using unions during a local search

Let *T* be the current candidate solution during a local search, and *U* the set of unions of *T* by applying Algorithm 3 while traversing the tree in Affine-DO. If a new candidate tree *S* is accepted during the local search, then update *U* using the direction for the best subdivision vertex computed for *S* (i.e. the one that bounds *S* with the lowest length). By maintaining this set of unions, we can modify the TBR local search as in Algorithm 4, to join only edges that are incident in unions at short distance. We call this method Union–pruning

### Algorithm 4: Heuristic Union-pruning TBR. The threshold 1.17 parameter was experimentally tuned

#### Building the initial trees

The Wagner algorithm is a basic procedure to compute an initial tree (Algorithm 5). We modify this procedure in two ways.

### Algorithm 5: The Wagner algorithm for initial tree building

#### 

##### Union–pruning.

Unions can be used to efficiently prune candidate trees during the Wagner algorithm by maintaining the union set of the tree *T* in Algorithm 5, and treat each leaf to be added as a union of its own. Then use Algorithm 4 to guide the join step in Algorithm 5.

#### 

##### Addition sequence

The initial sequence *L* in Algorithm 5 is typically randomized, assigning equal probability to each permutation. This algorithm is known as Random Addition Sequence (RAS). The randomization of L is used to obtain multiple starting points for local searches. We have explored the following variation successfully: 

1. Compute a Minimum Spanning Tree (MST) of *L* (i.e. the set of leaves).

2. Traverse *L* using a BFS. The order in which we visit the elements of *L* is our initial addition sequence *Q*(0).

3. To produce the *n*’th tree, produce the sequence *Q*(*n*) by flipping consecutive elements in *Q*(*n*−1) with probability 0.5.

We call this procedure MST-Wagner.

## Methods

We evaluated experimentally a number of algorithms for local searches under the GTAP. An experimental evaluation of this kind has three fundamental components: a selection of heuristics, implementation, and selection of data sets. The overall performance is compared with the length of the trees found by each method.

### Algorithms compared

We compared the following heuristic local searches, in all meaningful combinations. *TAP Computation*: Using Affine-DO in two variations, Exhaustive, and Non-exhaustive. *Building*: Wagner algorithm using RAS and MST addition sequences, and the Neighbor Joining (NJ) algorithm. The Wagner algorithm was executed with lookahead parameters of 1, 2, 4, and 10. *Neighborhood*: TBR and SPR (a subset of TBR). *Edge breaking order*: randomized, or in length decreasing order. *Join order*: randomized, or in ascending order based on the distance of the union that each edge belongs to. In the second case, the Union-pruning strategy was used to filter candidates. *Sector and reroot diameters*: 2, 3, 5, and infinity (i.e. no sector). The rerooting order followed a breadth first search (BFS) order, around the broken edge. The sector and reroot diameters were selected to match the simulation size (50 leaves). *Simulated annealing*: using initial temperatures of 2, 5, and 10, and coefficients of 12, 50, 250, and 500. The values were selected experimentally as a good sample of the performance variation observed by the authors in real GTAP problems.

For the edit distance parameters we tested the following combinations of substitution, indel, and gap opening parameters [total gap cost = gap opening + (length × indel)]: (1, 1, 0), (1, 2, 0), (2, 1, 1), (3, 1, 2). In our experience, these parameters encompass enough variation in the GTAP, while maintaining a limited number of combinations with the algorithms. In total, 34 combinations of build algorithms and distance functions were tested. For the refinement step, a total of 208 combinations of algorithms and edit distance functions were tested.

### Implementation

We implemented the algorithms under comparison in the Objective CAML and C programming languages. All the algorithms are available in the author’s computer program POY version 4 [9]. The functions are highly optimized for performance.

### Data sets

To generate the instance problems, we simulated sequences using DAWG 1.1.1 [36] with insertions and deletions following a power law distribution. The simulations followed random binary trees of 50 leaves comprising all the combinations of the parameters listed in Table 1. This tree size was chosen to be both tractable and realistic in size without biasing trees to any particular shape. The indel and branch parameters also were chosen to be similar to what is seen in empirical data sets. These produced a total of 30 independent simulations. Each simulation was analyzed independently with 100 repetitions for each randomized algorithm. NJ was tested only once, as our implementation is deterministic. An initial exploration with 300 repetitions showed no significant difference compared to 100 repetitions. In total, 102,000 builds, and 624,000 refinements were performed. Due to the large number of simulations and local searches performed, we will concentrate on a reduced set of cases that represent the overall patterns observed.

**Table 1:** Simulation parameters

**Parameter**	**Values Evaluated**
Substitution Rate	1.5
Average Branch Length	0.1,0.2,0.3,*∞*
Max. Gap	1,2,5,10,15
Root Sequence Length	500

All combinations of parameters were employed to generate the test data sets. The branch length variation equals the average branch length.

## Results and discussion

This section begins with the difference in performance between the Exhaustive (E) and the Non-exhaustive (NE) algorithms, which can be applied in conjunction with any other search strategy. It continues with a comparison of the build algorithms, and the refinement algorithms. Finally, we compose the results in a simple local search heuristic which we compared with the previous best heuristic on a real dataset.

### 

#### Exhaustive and non-exhaustive algorithms

**Figure 5:**
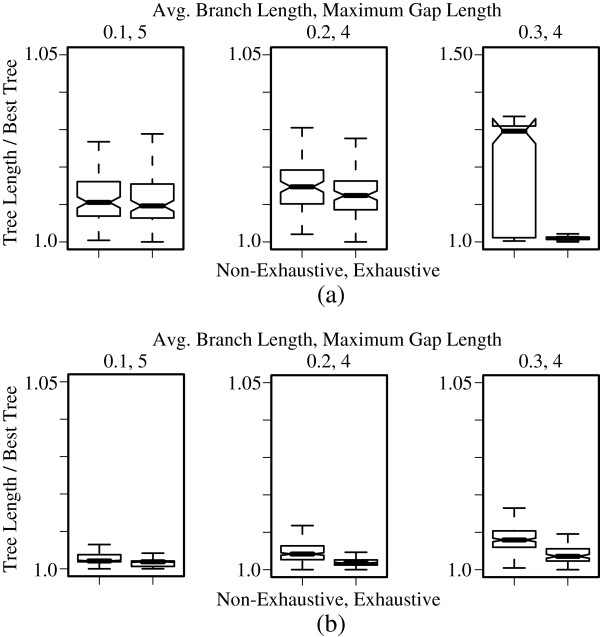
**Tree building algorithm comparison: NE vs E.** Comparison of the Non-Exhaustive (NE), and Exhaustive (E) TAP approximation algorithms in tree building (Figure a), and TBR (Figure b). The patterns showed were observed in most of the combinations of simulation, algorithm, and edit distance parameters. **a.** Tree building using the Wagner algorithm. In every case, E outperformed NE, but the difference is not significant. However, as the branch lengths increased, the performance of the NE algorithm showed high variability (right), making E highly competitive for all distance functions with average branch length 0.3. **b.** Refinement using Union-pruning with NE and E. In this case, for almost every combination of algorithm, simulation, and distance function, E produce significantly shorter trees.

In the build step (Figure 5), the difference between E and NE is small for all equivalent algorithms with branch lengths of 0.1 and 0.2 (Figure 5a, left and center). The most striking difference, however, occurs for branch length 0.3 (Figure 5a, right), where NE shows an expected tree length 50% higher than that of E. Such extreme variation shows a strong dependence on the root location when branch lengths make sequences close to random relative to each other.

For the TBR step, E significantly outperforms NE, with better minimum and expected scores (Figure 5b). This pattern was observed for every combination of algorithm, simulation, and edit distance parameters. In the following two sections, we concentrate on the results obtained using the E algorithm. The same general patterns were observed with NE, but with less competitive tree scores.

### 

#### Initial tree building

**Figure 6:**
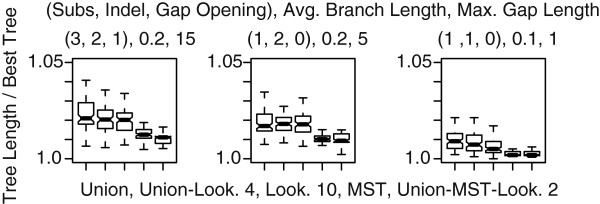
**Tree building algorithm comparison.** Comparison of initial tree build algorithms. *Union* is the Wagner algorithm + RAS + Union-pruning. *Union - Look. 4* is the Wagner algorithm + RAS + Union-pruning + Lookahead of at most 4 trees. *Look. 10* is the Wagner algorithm + RAS + Lookahead of at most 10 trees. *MST* is the Wagner algorithm + MST sequence, but no Union-pruning. *Union-MST-Look. 2* is the Wagner algorithm + MST sequence + Union-pruning + Lookahead of at most 2 trees.

The initial tree building algorithms fall into two main groups: algorithms with RAS, and algorithms using MST. In all cases, MST produced significantly shorter trees (Figure 6). The use of higher lookahead parameters did not produce consistent improvements in the resulting trees, while the use of the Union-pruning algorithm did significantly improve the expectation, and the minimum tree cost for branch lengths 0.1 and 0.2. For long branch lengths, however, no significant improvement was observed.

Neighbor joining produced trees of highest score among all the algorithms for all parameters (i.e. the worst, between 10% and 20% higher). We do not present it in the graphs as it would make the more subtle differences between other algorithms difficult to observe. Overall, the most important improvement occurs with the MST addition sequence in first place, followed by the use of the Union-pruning strategy in second. Nevertheless, we will see in the next section that the use of the MST algorithm remains limited.

### 

#### Refinement

To evaluate the TBR refinement experimentally, we must produce an initial tree. Although MST showed better results than RAS, we found that in almost every instance TBR failed to improve the MST trees. At the end, RAS + TBR would always find better trees than MST + TBR. For this reason, we used the second best method to construct the initial trees: RAS using Union-pruning.

The refinement comparison can be divided in two groups: 1.) a comparison between basic TBR using Union-pruning, and branch length sorting, and 2.) the comparison of different algorithms using the best combination among those in 1.

##### Union-pruning and branch length sorting

**Figure 7:**
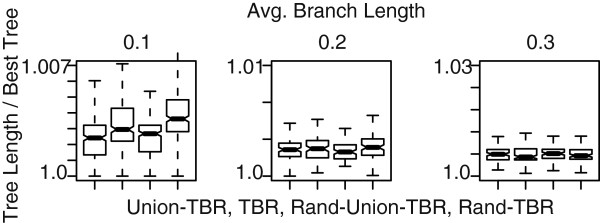
**Tree search algorithm comparison.** Comparative performance of Union-pruning, and branch length sorting, with randomized algorithms in TBR. *Union-TBR* is the length sorted edge break + Union-pruning. *TBR* is length sorted edge break + randomized edge break and edge join ordering. *Rand-Union-TBR* is a randomized edge break + Union-pruning. *Rand-TBR* is randomized edge break and edge join.

The behavior of TBR with Union-pruning and branch length sorting is presented in Figure 7, the Union-pruning algorithm produced significantly better trees, both in the minimum and expected scores. This advantage disappears as sequences diverge to close to random (branch length of 0.3) (Figure 7 left to right). Branch length sorting had a small positive impact, but not significative.

The results match our expectation: the Union-pruning algorithm can positively guide the search with better taxon sampling. We have observed this behavior in real data sets, where new terminals some times *speedup* the local search. This somewhat counter-intuitive behavior is likely due to the structured nature of phylogenetic data. The addition of new terminals increases the data support for subtrees, thereby increasing the cost penalty when these groups are violated. Union-pruning takers advantage of this in creating unions from larger sets of taxa, hence containing more information. As the data become less structured (approaching random as mentioned above) the effect disappears.

##### Local search strategy

Beyond the use of Union-pruning, and Exhaustive TAP estimation, the differences among the algorithms compared are not significant (Table 2). Although in general Sectorial finds the shortest tree with highest frequency, the difference is typically less than two length units, compared to the second best algorithm. In general, the algorithm with the best mean is BFS, but again, not significative. However, due to the algorithm design, BFS is the fastest of all.

**Table 2:** Minimum and average tree score comparison among algorithms using Union-pruning and Exhaustive TAP estimation

**Gap Len.**	**Edition Distance**			**TBR**		**Sectorial**		**BFS**		**Annealing**	
	**Subst.**	**Indel**	**GO**	**Min.**	**Avg.**	**Min.**	**Avg.**	**Min.**	**Avg.**	**Min.**	**Avg.**
1	1	1	0	7190	7222.75	**7186**	7221.188	7190	**7220.969**	7198	7230.802
	1	2	0	8410	8437.76	8405	**8429.812**	**8406**	8436.865	8416	8457.24
	2	1	1	**14022**	14111.76	14032	14107.58	**14022**	**14096.88**	14031	14144.56
	3	1	2	20089	20236.07	20118	20303.64	**20062**	**20221.83**	20172	20373.85
2	1	1	0	6680	6702.115	**6674**	**6697.76**	6676	6699.719	6687	6713.854
	1	2	0	7969	7992.562	7963	**7989.333**	7969	7990.583	**7967**	8005.479
	2	1	1	12994	13040.67	**12978**	13034.80	12981	**13030.21**	13001	13074.80
	3	1	2	18603	18690.26	**18588**	18716.78	18589	**18678.82**	18629	18785.17
4	1	1	0	**7164**	7190.719	**7164**	**7186.323**	7166	7188.062	7176	7208.594
	1	2	0	**8684**	8719.552	**8684**	**8714.406**	8682	8716.677	8698	8751.26
	2	1	1	**13586**	13652.25	13590	13658.08	13592	**13646.89**	13601	13694.72
	3	1	2	19148	19291.41	19149	19344.61	**19113**	**19283.66**	19209	19448.12
5	1	1	0	7049	7077.542	**7043**	7074.229	7049	**7073.729**	7057	7092
	1	2	0	8692	8716.01	**8683**	8715.5	8688	**8711.104**	8690	8730.646
	2	1	1	**13329**	13389.48	13334	13394.16	13336	**13387.41**	13363	13429.17
	3	1	2	18876	18983.53	**18861**	19027.35	18870	**18974.93**	18930	19091.70
10	1	1	0	7149	7181.74	**7141**	**7174.938**	7145	7176.719	7163	7200.5
	1	2	0	8965	9002.677	**8944**	8993.438	8948	**8992.656**	8979	9020.635
	2	1	1	13200	13271.72	13199	13277.82	**13195**	**13266.54**	13235	13320.24
	3	1	2	**18395**	18557.96	18423	18630.5	18402	**18549.86**	18470	18648.79
15	1	1	0	7162	7194.01	7160	7194.531	**7159**	**7190.542**	7182	7216.719
	1	2	0	9151	9196.552	**9142**	9192.125	9147	**9191.344**	9151	9228.146
	2	1	1	13168	13230.11	13164	13231.83	**13155**	**13217.84**	13186	13271.46
	3	1	2	18194	18350.44	18234	18415.64	**18166**	**18335**	18290	18484.11

The differences observed are not significant. All the simulations shown have branch length 0.3, but similar patterns were observed for branch lengths 0.1 and 0.2. The minima across each row is in bold.

### Overall performance

Based on the previous experiments, we prefer a heuristic local search strategy that consists of the following steps: build initial trees using RAS guided by Union-pruning, followed by a refinement step consisting of TBR using the three directional heuristics, Exhaustive TAP, Union-pruning, and cutting edges according to descending lengths. We compared this algorithm (implemented in POY version 4), with that of POY version 3 which uses a one directional algorithm, with randomized TBR steps [19,37]. Due to limitations in POY version 3’s implementation, we only compare an edition distance with substitution parameter 1, indel parameter 1, and gap opening parameter 0. Due to the implementation limitation, MSAM was not included in the comparison.

For this comparison, a random subset of 100 published anurans [38] was analyzed. The data set includes 12S rRNA, tRNA valine, 16S rRNA, and fragments of cytochrome b, rhodopsin, tyrosinase, 28S rRNA, and RAG 1, and a small set of 38 morphological, non-additive characters (i.e. Hamming distance model).

**Figure 8:**
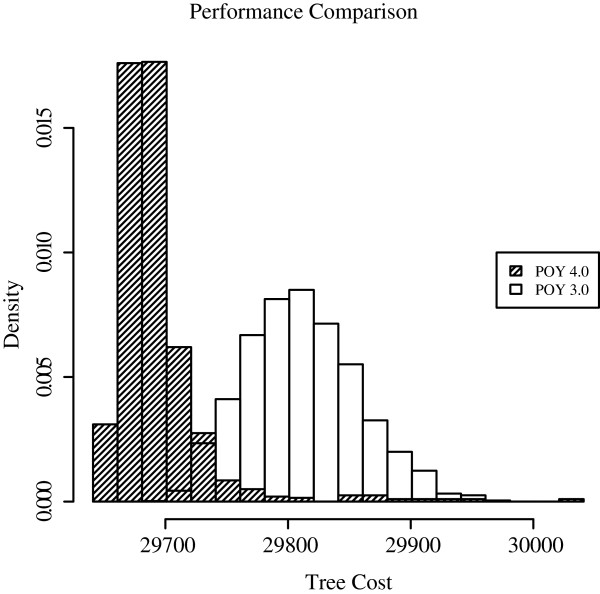
**Comparison of new algorithms vs old algorithms.** Density histogram of the frequency of occurrence of different tree scores in POY version 3 and version 4 for the example data set.

To compare the performance of POY version 3 and version 4, we executed 1000 independent repetitions consisting of 1 build, followed by refinement, and reported the resulting tree score. This procedure can be executed in POY 3 with the command: poy ‐replicates 1 ‐seed ‐1 ‐maxtrees 1 ‐nooneasis ‐minterminals 0 ‐terminalsfile ranNamesPH.txt ⋆.fas ⋆.ss. The score of the trees found by each program were plotted in a density histogram (Figure 8). The results show that one repetition of our new heuristic in POY version 4 outputs a tree which is expected to belong to the top 15% of the best trees found by this very simple search strategy. To expect a tree within the same percentile using the old heuristic, it would be necessary to run more than 2000 local searches. It follows that through combination and speed and efficiency, the new heuristic is more than 2000 times faster than the previous heuristic of POY 3.

### Discussion

We described and implemented new heuristics for the GTAP. We have shown that they find better solutions than previous approaches. We found that a number of conditions affect the fit of the heuristic to the problem: long branch–length data sets can be better analyzed with Sectorial Search instead of the Union-pruning, while Union-pruning yields excellent results in medium, and short branch lengths. Exhaustive-TBR yields the best results overall and should always be preferred. Although the MST algorithm yields better initial results than RAS, it is not preferable in the long run, and a small number of local searches should never be used to produce reliable results. It remains to be explored the quality of the numerous meta–heuristics available in the literature. It is now possible to explore them using a more efficient local search strategy.

## Conclusions

We described new strategies that can be composed to produce a powerful local search strategy for the Tree Alignment Problem. The results showed that our methods improve on the best existing local search heuristics by more than three orders of magnitude.

In general, the Exhaustive–TBR refinement strategy should always be used, while Union-pruning should only be preferred if dense taxon sampling or short branch lengths are expected. Moreover, although the MST build strategy yields better results than the traditional Wagner build, the former should not be preferred in real analyses since it tends to produce less competitive trees after the refinement step.

It is difficult to predict the performance of other high level heuristics applied to the GTAP. Strategies such as Sectorial Search, and Tree Fusing should be effective. However, Divide and Conquer techniques such as DCM-3 may have a more limited application, unless used in the spirit of Sectorial Search. Given that phylogenetic analysis under MP shows a simplified setting compared to other optimality criteria, it is our opinion that metaheuristics such as Simulated Annealing have limited applicability in the join estimation of tree and alignments for all optimality criteria, and novel strategies are needed to successfully scale to larger problem sizes. Nevertheless (unless *P*=*NP*), all these strategies will belong to the heuristic realm, and further experimental efforts will be required.

Affine-DO, Union–pruning, and Exhaustive–TBR are some of the algorithms that we have implemented in the computer program POY version 4 [9]. The algorithms and their implementation have had a significant impact in the biology community interested in different approaches to joint tree and phylogeny reconstruction. By using better algorithms, algorithm engineering, and better parallel strategies, POY version 4 is three orders of magnitude faster than its predecessor. The concepts, and desirable properties of this implementation should be extended to other phylogenetic inference criteria, to broaden its usability, and better serve the research purposes set for the software package.

## Competing interests

The authors declare that they have no competing interests.

## Authors’ contributions

The authors contributed equally to this work. Both authors read and approved the final manuscript.
